# Prostaglandin PGE_2 _at very low concentrations suppresses collagen cleavage in cultured human osteoarthritic articular cartilage: this involves a decrease in expression of proinflammatory genes, collagenases and *COL10A1*, a gene linked to chondrocyte hypertrophy

**DOI:** 10.1186/ar2273

**Published:** 2007-08-07

**Authors:** Elena V Tchetina, John A Di Battista, David J Zukor, John Antoniou, A Robin Poole

**Affiliations:** 1 Shriners Hospitals for Children, Departments of Surgery and Medicine, McGill University, 1529 Cedar Avenue, Montreal, Quebec H3G 1A6, Canada; 2 Division of Rheumatology, Department of Medicine, 687 Pine Avenue West, Montreal, Quebec H3A 1A1, Canada; 3 Jewish General Hospital, McGill University, 3755 Cote St. Catherine Road, Montreal, Quebec H3T 1E2, Canada; 4 Department of Surgery, 687 Pine Avenue West, McGill University, Montreal, Quebec H3A 1A1, Canada; 5 Genetics Department, Institute of Rheumatology, Russian Academy of Medical Sciences, Kashirskoye shosse 34A, Moscow 115522, Russia

## Abstract

Suppression of type II collagen (COL2A1) cleavage by transforming growth factor (TGF)-β2 in cultured human osteoarthritic cartilage has been shown to be associated with decreased expression of collagenases, cytokines, genes associated with chondrocyte hypertrophy, and upregulation of prostaglandin (PG)E_2 _production. This results in a normalization of chondrocyte phenotypic expression. Here we tested the hypothesis that PGE_2 _is associated with the suppressive effects of TGF-β2 in osteoarthritic (OA) cartilage and is itself capable of downregulating collagen cleavage and hypertrophy in human OA articular cartilage. Full-depth explants of human OA knee articular cartilage from arthroplasty were cultured with a wide range of concentrations of exogenous PGE_2 _(1 pg/ml to 10 ng/ml). COL2A1 cleavage was measured by ELISA. Proteoglycan content was determined by a colorimetric assay. Gene expression studies were performed with real-time PCR. In explants from patients with OA, collagenase-mediated COL2A1 cleavage was frequently downregulated at 10 pg/ml (in the range 1 pg/ml to 10 ng/ml) by PGE_2 _as well as by 5 ng/ml TGF-β2. In control OA cultures (no additions) there was an inverse relationship between PGE_2 _concentration (range 0 to 70 pg/ml) and collagen cleavage. None of these concentrations of added PGE_2 _inhibited the degradation of proteoglycan (aggrecan). Real-time PCR analysis of articular cartilage from five patients with OA revealed that PGE_2 _at 10 pg/ml suppressed the expression of matrix metalloproteinase (MMP)-13 and to a smaller extent MMP-1, as well as the proinflammatory cytokines IL-1β and TNF-α and type X collagen (COL10A1), the last of these being a marker of chondrocyte hypertrophy. These studies show that PGE_2 _at concentrations much lower than those generated in inflammation is often chondroprotective in that it is frequently capable of selectively suppressing the excessive collagenase-mediated COL2A1 cleavage found in OA cartilage. The results also show that chondrocyte hypertrophy in OA articular cartilage is functionally linked to this increased cleavage and is often suppressed by these low concentrations of added PGE_2_. Together these initial observations reveal the importance of very low concentrations of PGE_2 _in maintaining a more normal chondrocyte phenotype.

## Introduction

Osteoarthritis (OA) is a systemic condition that can affect single or multiple joints and involves degenerative changes in the articular cartilage, remodeling of subchondral bone and limited synovial inflammation [1]. Osteoarthritic changes in articular cartilage involve progressive proteolytic degradation of its extracellular matrix, composed mainly of type II collagen (COL2A1) and aggrecan, eventually leading to a loss of the cartilage. This involves phenotypic hypertrophy-related changes in chondrocytes, such as the production of type X collagen (COL10A1) (hypertrophy marker), and the upregulation of collagenase matrix metalloproteinase (MMP)-13, as is seen in the fetal growth plate [1-3].

Joint inflammation in OA causes an increased synthesis of cyclooxygenase (COX)-2-dependent prostaglandins (PGs), which sensitize peripheral nociceptor terminals and produce localized sensitivity to pain [4]. Non-steroidal anti-inflammatory drugs and specific COX-2 inhibitors are therefore most widely used as painkillers to inhibit prostaglandin production by COX. Prostaglandins, especially PGE_2_, the major PG synthesized by cartilage [5], are spontaneously released by OA cartilages in amounts 50-fold higher than in normal cartilage and 18-fold higher than in normal cartilage stimulated by cytokines [6]. Strong upregulation of COX-2 expression in arthritic synovial membranes and cartilage has led to the suggestion that the selective inhibition of COX-2 may result in an amelioration of arthritic conditions [7].

However, PGE_2_, generated by chondrocytes, has been shown to be physiologically important for maintaining cartilage homeostasis [6]. Because COX-2 is expressed physiologically in some tissues such as glomeruli and cortex, it may have an anti-inflammatory effect [4,8]. It can be protective in OA articular cartilage because it inhibits the expression of IL-1β-induced collagenase and stromelysin in human and animal synovial fibroblasts [9,10], and stimulates collagen and proteoglycan synthesis [11-13] and chondrocyte proliferation [11,14].

Moreover we showed recently that the growth factor TGF-β2 is capable of suppressing chondrocyte hypertrophy and differentiation, collagenase expression, collagen cleavage and the expression of proinflammatory cytokines in cultured OA articular cartilages [3]. This is accompanied by the upregulation of prostaglandin E synthase-1 (PGES-1) expression and PGE_2 _release. Here we test the hypothesis that PGE_2 _alone can inhibit collagen degradation in OA articular cartilage, and provide evidence in support of it.

## Materials and methods

### Patients

Human femoral condylar cartilages were obtained at total knee arthroplasty from 19 patients (*n *= 4 men, mean age 73.7 ± 6.4 years, range 66 to 80 years; *n *= 15 women, mean age 77.2 ± 11.3 years, range 50 to 90 years) with OA diagnosed in accordance with the criteria of the American College of Rheumatology [15]. The study was approved by McGill University Ethics Review Board.

### Isolation and preparation of cartilage

Femoral condylar articular cartilages were isolated and prepared for culture as described previously [3,16]. All cartilages exhibited macroscopic articular surface differences from normal cartilages. To generate sufficient cartilage to perform each of these analyses on a patient, all of the available articular cartilage from each patient, regardless of the degree of degeneration (Mankin grades 4 to 12), was used, as described previously [3,17,18]. In brief, OA articular cartilages were washed three times with DMEM (Gibco BRL, Life Technologies, Grand Island, NY, USA) containing 20 mM HEPES (4-(2-hydroxyethyl)-1-piperazine ethanesulfonic acid) buffer pH 7.4 (Gibco BRL), 45 mM NaHCO_3_, 100 units/ml penicillin, 100 μg/ml streptomycin and 150 μg/ml gentamycin sulphate. Full-depth cartilage slices from a single site, about 20 mm × 20 mm, were cut vertical to the articular surface and then into cubes of about 2 mm × 2 mm. Five to seven cubes were randomly obtained and wet weights of about 60 mg were distributed in each culture well (48-well Costar 3548 plate; Corning Inc., Corning, NY, USA). Samples were maintained before culture for 48 hours at 37°C in 1 ml per well of medium A in 95% air/5% CO_2_.

In these and our previous studies of these human knee OA cartilages we used a standard sampling procedure [8,19]. OA femoral condylar cartilages show variations in cartilage thickness and various degrees of degradation between weight-bearing and non-weight-bearing regions. These result in variation between samples, although we do our best to finely chop, mix and randomly distribute the tissue in our culture wells from a given joint and person. In spite of this variability we observed a significant difference in collagen cleavage activity between PGE_2_-treated cartilage explants and the untreated controls.

The amount of OA cartilage, which is always macroscopically different from normal, is always limiting. We did not use Mankin grading because the degeneration is very variable within the joint and, as in the previous studies, we had to use all the cartilage available from each patient to be able to perform these analyses, otherwise it would not have been possible to conduct the experiments. This mixture of finely chopped cartilages therefore represents different degrees of degeneration existing within a given joint, from Mankin grade 4 to grade 12.

### Cartilage explant culture

Media were changed after 48 hours (day 0) and thereafter were replaced every 4 days. Final concentrations of 1 pg/ml to 10 ng/ml PGE_2 _(Sigma Chemical Co., St. Louis, Mo, USA) or 5 ng/ml TGF-β2 (R&D Systems, Minneapolis, MN, USA) were freshly added to medium A from day 0 at each medium change. The cartilage (triplicate cultures for each analytical point) was cultured for a total of 16 days and analyzed at day 16 for COL2A1 cleavage by collagenases and proteoglycan release. The conditioned media were collected every 4 days at each medium change from day 4 to day 16 and stored at -20°C until analyzed. For analyses of gene expression, separate cultures were maintained for up to 48 hours and analyzed as described below.

### ELISA assays of collagenase-cleaved type II collagen

The OA cartilage explants from day 16 of culture were digested and extracted with α-chymotrypsin to solubilize denatured collagen including the carboxy-terminal neoepitope COL2–3/4C short (C1,2C) epitope generated by the cleavage of COL2A1 by collagenase. This was measured as described previously in α-chymotrypsin extracts and conditioned media by ELISA [17,18]. Total amount of cleavage neoepitope in cartilages and media was calculated by summation of the data from each medium change and cartilage analysis. Results were expressed as pmoles of epitope per mg wet weight of cartilage, based on a molecular mass of the standard peptide epitope of 608 Da.

### Determination of proteoglycan content and release

This was determined in cartilage extracts and conditioned media as sulfated glycosaminoglycans (GAGs), which is primarily a measure of proteoglycan aggrecan content, using a modification of the colorimetric 1,9-dimethylmethylene blue dye-binding assay [20]. Cumulative proteoglycan release (GAG in the medium) and its content in cartilages (GAG in the cartilage) were expressed as mirograms of GAG per milligram wet weight of cartilage. GAG release into medium was represented as a percentage of the total cumulative GAG in the cartilage plus cumulative GAG release into medium. This provides an accurate measure of release, accounting for the marked variation in cartilage GAG content in OA cartilage [18,21].

### Quantification of PGE_2 _release

PGE_2 _concentrations were determined in the control undiluted conditioned media collected at each medium change, which were then pooled for analysis with a commercially available competitive ELISA kit (Cayman Chemical Company, Ann Arbor, MI, USA,) in accordance with the manufacturer's instructions. Results were expressed in picograms per milligram wet weight of cartilage. PGE_2 _standards provided with the kit were diluted with DMEM.

### Total RNA isolation

Total RNA isolation for the detection of gene expression was based on methodology previously described [3]. This was isolated from articular cartilage explants or isolated chondrocytes after up to 96 hours in culture as indicated in the Results section. Fresh cartilage tissue or cells in solution D (4 M guanidine isothiocyanate, 25 mM sodium citrate pH 7.0, 0.5% laurylsarcosine, 0.1 M 2-mercaptoethanol) were immediately frozen in liquid nitrogen and kept at -80°C until all samples had been collected. Samples were defrosted at 21–23°C and then vortex-mixed vigorously for 30 minutes. The debris was removed by centrifugation at 5,000 *g *for 10 minutes at 4°C. Proteins and nucleic acids in the supernatant were precipitated with 1 volume of propan-2-ol overnight at -20°C. The precipitate was removed at 10,000 *g *for 20 minutes at 4°C and resuspended in digestion buffer (10 mM Tris-HCl pH 8.0, 5 mM ethylenediaminetetraacetic acid, 1% SDS, with 2 mg/ml Proteinase K (Gibco BRL)) and incubated at 50°C until the pellet disappeared. After extraction with a mixture containing 1 volume of phenol, 0.2 volumes of chloroform and 0.1 volume of 2 M sodium acetate pH 4.0, the aqueous phase was recovered by centrifugation (10,000 *g *for 30 minutes at 4°C). An equal volume of 70% ethanol was added to each sample aqueous phase and loaded on an RNeasy spin column (Qiagen, Valencia, CA, USA). Further RNA purification was performed with an RNeasy kit (Qiagen) in accordance with the manufacturer's instructions.

### Reverse transcriptase reaction

The reverse transcriptase reaction was performed with total RNA isolated from articular cartilage explants and SuperScript TMII H^- ^Reverse Transcriptase as recommended by Gibco BRL-Invitrogen, (Burlington, ON, Canada) and as described previously [3].

### Real-time quantitative PCR

Preformed primers and probes for the TaqMan assay (Applied Biosystems, Foster City, CA, USA) of human genes used in this study were MMP-13 Applied Biosystems, TaqMan Gene Expression Assays, accession no. Hs00233992_m1), COL10A1 (Hs00166657_m1), MMP-1(Hs00233958_m1), IL-1β (Hs00174097_m1) and TNF-α (Hs00174128_m1). Glyceraldehyde-3-phosphate dehydrogenase (GAPDH), β-actin, 18s RNA and cyclophilin were examined as endogenous controls. GAPDH has been chosen for the final data presentation because it gives the lowest variation.

Quantification of gene expression levels of mRNA was performed with a 7500 Real-time PCR System (Applied Biosystems). After treatment with 1 μl of RNase H, 1 μl of reverse transcription product was subjected to real-time PCR in a 25 μl total reaction mixture containing 12.5 μl of TaqMan Universal PCR Master Mix (Applied Biosystems), 900 nM sense and antisense primers, 50 nM probe, and template cDNA. After a single step of 50°C for 2 minutes and initial activation at 95°C for 10 minutes, reaction mixtures were subjected to 40 amplification cycles (15 seconds at 95°C for denaturation, and 1 minute of annealing and extension at 60°C).

By using a sequence detection system the threshold cycle (*C*_*t*_) was determined at which the exponential amplification of PCR products began. After PCR, dissociation curves were generated with one peak, indicating the specificity of the amplification. A threshold cycle (*C*_*t *_value) was obtained from each amplification curve by using SDS V 1.3 software provided by the manufacturer.

Relative mRNA expression was determined with the ΔΔ*C*_*t *_method, as detailed by manufacturer guidelines (Applied Biosystems). A Δ*C*_*t *_value was calculated by subtracting the *C*_*t *_value for the housekeeping gene GAPDH from the *C*_*t *_value for each sample. A ΔΔ*C*_*t *_value was then calculated by subtracting the Δ*C*_*t *_value of the control (unstimulated cartilage) from the Δ*C*_*t *_value of each treatment. Fold changes compared with the control were determined by raising 2 to the power -ΔΔ*C*_*t*_; that is, 2^-ΔΔ*Ct*^. Each PCR was performed in duplicate on three separate occasions for each independent experiment. Three 'no template' controls were consistently negative for each reaction.

To avoid variation in efficiency between experiments, all samples of the same cartilage were simultaneously subjected to reverse transcription, and all samples of cDNA were simultaneously amplified by real-time PCR.

The relative quantification protocol for evaluating gene expression with real-time reverse transcription-mediated PCR is based on the comparison of gene expression level in the treated cartilage explants and untreated cartilage control samples, both of which are normalized to the GAPDH expression level. The expression level of each control is set to 1. We therefore show only one control bar for all the genes measured in the set.

The deviations in treated samples represent variations in gene expression in triplicates of cultured cartilage from each individual. Real-time PCR reaction was performed in duplicate for each cartilage sample.

### Statistical analysis

Quantitative data are expressed as means ± SD. Assays were run in at least triplicate. A normality test showed that data were distributed in accordance with a Gaussian distribution curve. To analyze treatment effects, Mann–Whitney and paired *t*-test analyses were used. *P *< 0.05 was considered significant.

## Results

### Inhibition of collagenase activity by PGE_2_

**Figure 1:**
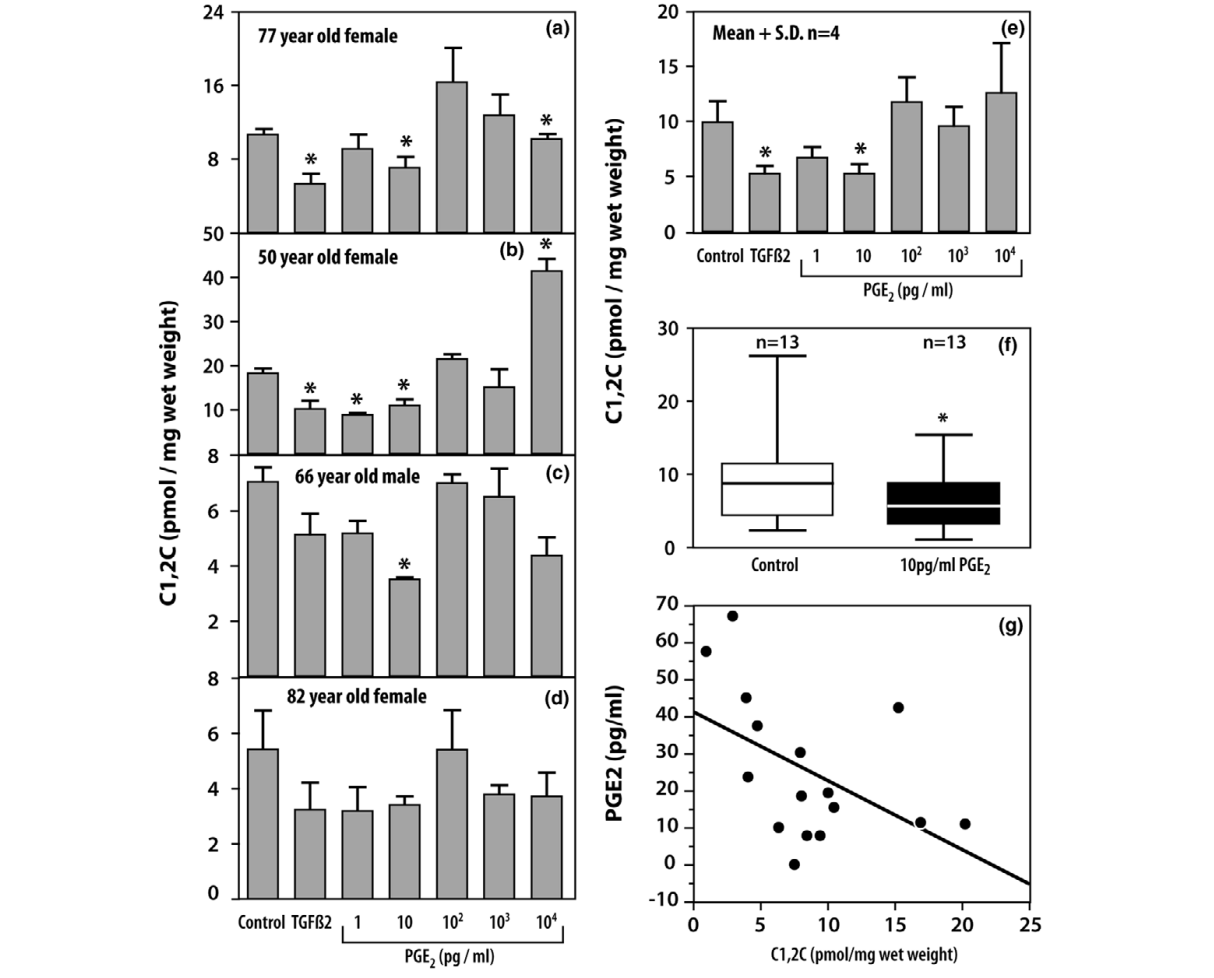
Inhibition of collagen cleavage by prostaglandin E_2 _(PGE_2_) and transforming growth factor (TGF)-β2. **(a–d) **The effect of 5 ng/ml TGF-β2 and PGE_2 _at various concentrations (1 pg to 10 ng/ml) on collagen cleavage in human osteoarthritic (OA) explants from patients of indicated age and gender; **(e) **means ± SDs for all four patients; **(f) **inhibition of type II collagen cleavage by collagenase in human OA articular explants by 10 pg/ml PGE_2_; **(g) **concentration relationship between PGE_2 _in control cultures and collagen cleavage. The average medium levels of PGE_2 _in the dose-dependent studies were as follows: 6.3 ± 1.2 pg/ml for the 77-year-old female (a); 13.9 ± 4.1 pg/ml for the 50-year-old female (b); 7.8 ± 2.1 pg/ml for the 66-year-old male (c); and 61.6 ± 7.6 pg/ml for the 82-year-old female (d). (f) Thirteen OA articular cartilage explants were cultured with (10 pg/ml PGE_2_) or without (control) for 16 days and collagen cleavage was evaluated by the accumulation of C1,2C neoepitope in the medium and chymotrypsin-derived cartilage extracts. (g) The relationship between PGE_2 _concentration and collagen cleavage in 16 OA explants that served as controls. Significant differences from the control (*P *< 0.05) are indicated by asterisks.

Our previous studies of cultured human OA articular cartilage explants have shown that conditioned media from cultures maintained with TGF-β2 for 16 days contained concentrations of PGE_2 _in the range 4 to 125 pg/ml. We used this information to determine whether the addition of exogenous PGE_2 _concentrations in this range would influence the cleavage of COL2A1 by collagenases in human OA explants. OA articular cartilage explants showed significant variability in the responsiveness to PGE_2_. Collagen cleavage was downregulated by exogenous PGE_2 _at concentrations as low as 0.1 pg/ml (data not shown) and 1 pg/ml in one of four patients (Figure 1b). However, in most examined cartilages and as shown by the group analyses, significant downregulation of collagen cleavage was observed at 10 pg/ml PGE_2 _in these four individual patients (Figure 1e) and in a larger cohort of 13 other patients (Figure 1f). A mean inhibition of 39.7% (range 33.5 to 49.8%) by 10 pg/ml PGE_2 _was observed and it was as effective as TGF-β2 (40.1%; range 26.9 to 49.4%). Interestingly, at higher concentrations (0.1 to 10 ng/ml) this inhibition was no longer seen. In one case, stimulation was observed at 10 ng/ml PGE_2 _(Figure 1b).

We observed an inverse relationship between PGE_2 _concentration and collagen cleavage activity in OA explant cultures that served as controls by plotting PGE_2 _concentration versus collagen cleavage in the control OA articular cartilage explants (Figure 1g). An increase in PGE_2 _levels in OA articular cartilage explants was accompanied by a linear (correlation coefficient *r *= -0.507; *P *= 0.044) decrease in collagen cleavage.

### Proteoglycan release in explants cultured with PGE_2_

**Figure 2:**
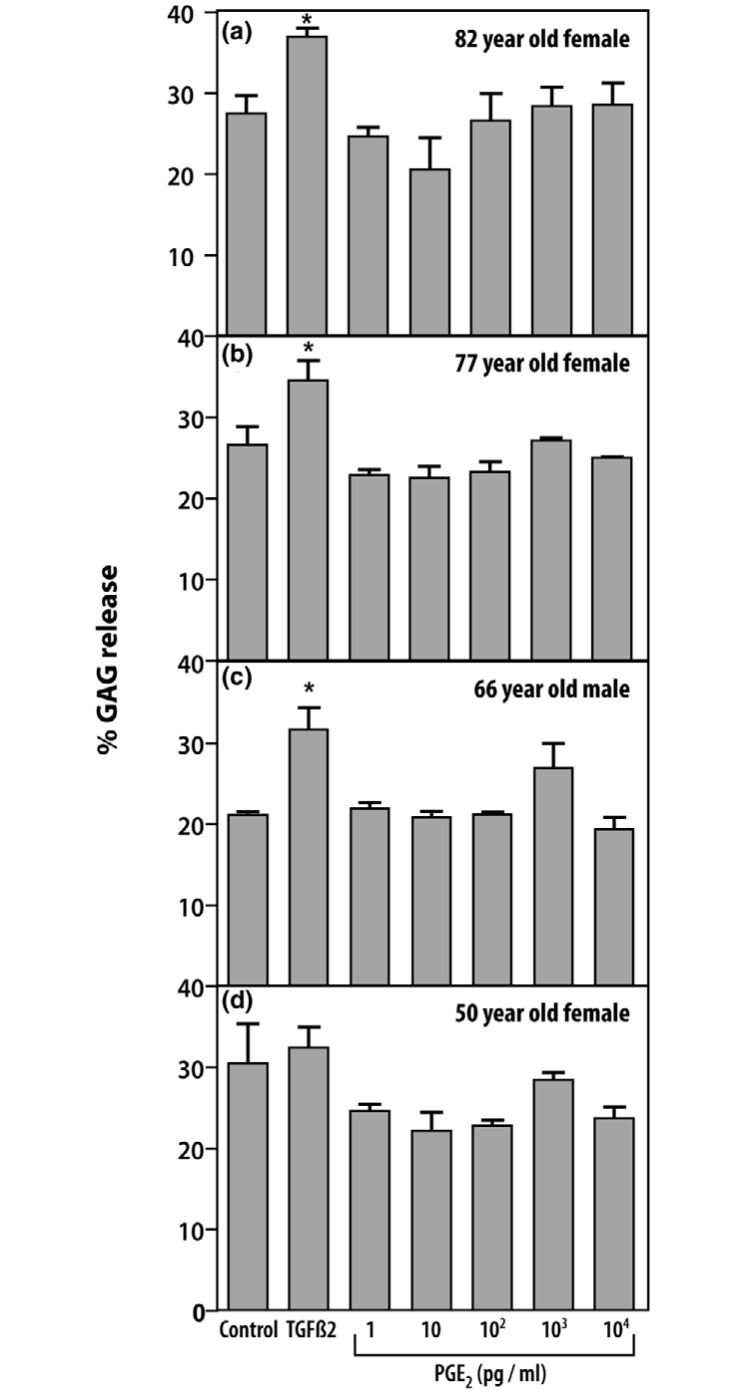
Prostaglandin E_2 _(PGE_2_) does not affect total proteoglycan release. **(a–d) **Percentage release of total proteoglycan (glycosaminoglycan (GAG) release) was measured in conditioned media of human osteoarthritic cartilage explants cultured with 5 ng/ml transforming growth factor-β2 or PGE_2 _at concentrations from 1 pg/ml to 10 ng/ml. The age and sex of each patient are indicated. Significant differences from controls (*P *< 0.05) are indicated by asterisks.

Downregulation of collagen cleavage was not accompanied by significant changes in GAG release in explants cultured in the presence of 10 pg/ml PGE_2 _(Figure 2). However, TGF-β2 significantly upregulated the release of GAG in three out of four cartilages examined.

### Alterations of gene expression in OA cartilage explants by PGE_2_

**Figure 3:**
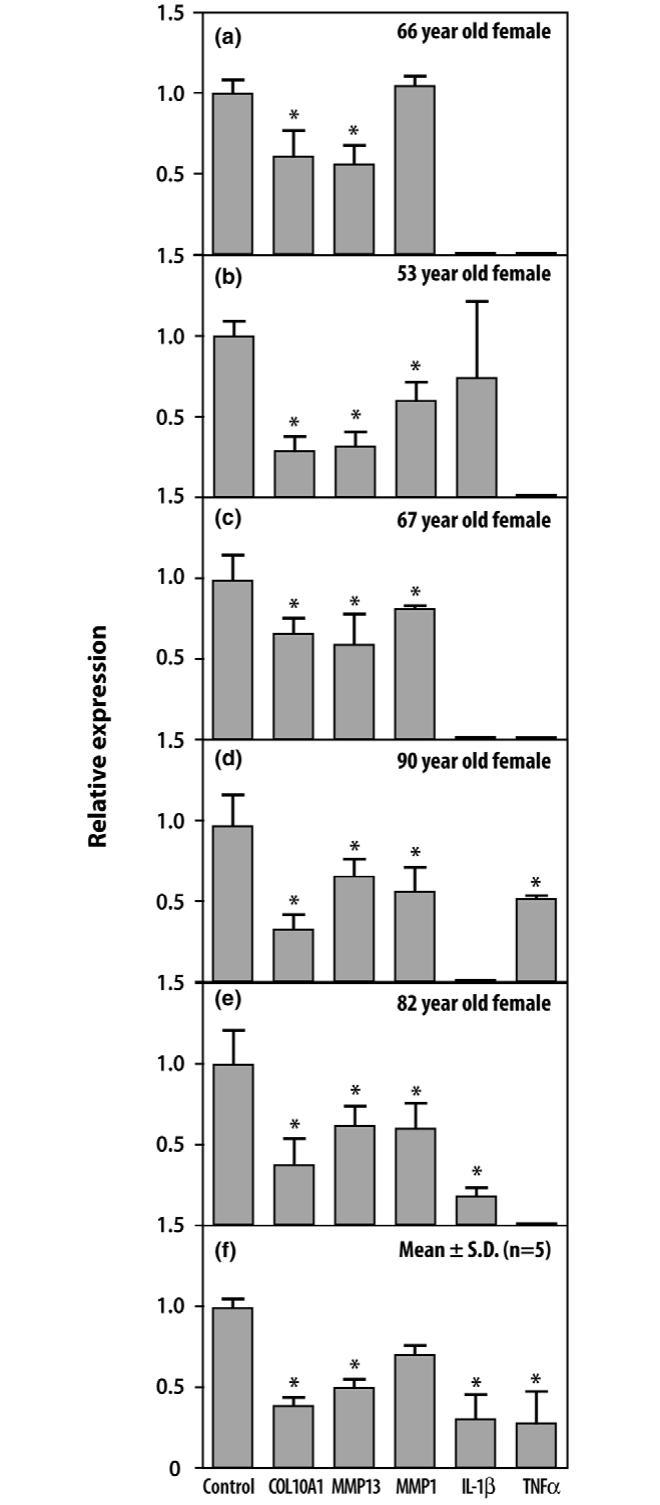
PGE_2 _downregulates the expression of genes responsible for collagen cleavage, chondrocyte hypertrophy and inflammation. Relative expression with reference to glyceraldehyde-3-phosphate dehydrogenase is shown compared with controls for genes in osteoarthritic cartilages determined by real-time PCR analyses in explant cultures at 24 hours cultured in the presence or absence (control) of 10 pg/ml prostaglandin E_2 _(PGE_2_). Control bars are shown as 1.0 as required for relative quantification with the real-time PCR protocol. Means ± SD for all five patients **(a–e) **are shown in **(f)**. Asterisks indicate significant differences from the control (*P *< 0.05). The age and sex of each patient are indicated. The average levels of PGE_2 _in the medium in the gene expression studies were as follows: 62.3 ± 6.1 pg/ml for the 66-year-old female (a); 7.2 ± 1.9 pg/ml for the 53-year-old female (b); 0 pg/ml for the 67-year-old female (c); 65.7 ± 7.3 pg/ml for the 90-year-old female (d); and 30.4 ± 7.6 pg/ml for the 82-year-old female (e).

In comparison with controls, OA explants from five patients cultured in the presence of 10 pg/ml PGE_2 _showed decreased expression of the genes related to chondrocyte hypertrophy, namely those encoding COL10A1 and MMP-13 (Figure 3). Expression of collagenase MMP-1 was significantly downregulated in four out of five patients. Cytokines were most strongly downregulated by prostaglandin and no expression of IL-1β (Figure 3a,c,d) or TNF-α (Figure 3a,b,c,d) was observed in explants cultured in its presence. However, expression of cyclin B2, caspase 3, TGF-β2, COX2 and PGES-1 was not significantly affected by 10 pg/ml PGE_2 _(data not shown). The data for the whole group are shown in Figure 3f. The variability of the residual expression level of genes such as those encoding IL-1 and TNF may result from the differences in cytokine expression associated with various disease states or activities.

## Discussion

COX-dependent prostaglandins have been implicated in various physiological processes, including male fertility, menstruation, ovulation, pregnancy and implantation, and in the pathological action of different inflammatory and neoplastic diseases, notably arthritis and cancer [4]. The potential pathological effects of PGE_2 _on articular cartilage have also been reported [7,22].

Here we present evidence to indicate that at very low concentrations (10 pg/ml) exogenous PGE_2 _is capable of selectively inhibiting excessive collagen cleavage seen in cultured human OA articular cartilage explants. This is accompanied by the downregulation of expression of the collagenases MMP-1 and MMP-13, the cytokines IL-1β and TNF-α, and COL10A1, the last of these being a marker of chondrocyte hypertrophy. Moreover, we observed an inverse relationship in control cultures (no added PGE_2_) between PGE_2 _content and COL2A1 cleavage. Together these results draw attention to the capacity of exogenous PGE_2 _to suppress collagen cleavage and PGE_2 _content.

It is unclear why very low exogenous concentrations of 10 pg/ml PGE_2 _can suppress collagen cleavage when cultures already contain up to 70 pg/ml PGE_2_. This may be related to the fact that PGE_2 _activity is unstable [23]. Thus, repeated application of PGE_2 _with each medium change may have been enough to decrease collagen cleavage. We do not know whether the PGE_2 _that we measured in cultures by immunoassay is active, but our results suggest that it is not.

The inhibitory effect of very low concentrations PGE_2 _in OA cartilage explants may help to explain the similar inhibitory effects produced by TGF-β_2 _that are accompanied by upregulation of PGE_2 _at the levels of gene expression and secretion [3]. The predominant inhibitory effect of PGE_2 _in OA cartilage explants supports the notion that our previously observed suppression of collagen cleavage by TGF-β_2 _may be the result of an autocrine/paracrine feedback loop involving the upregulation of the phospholipase A2/COX/PGES-1 axis and eicosanoid release [3]. The effect of added PGE_2 _and the interrelationships we observed in control cultures suggests an interesting dynamic in which PGE_2 _in the range 0 to 70 pg/ml released from OA specimens is associated with the suppression of collagen cleavage.

To obtain a more precise insight into the interrelationships between PGE_2 _and TGF-β2 effects in OA articular cartilage, activation of TGF-β2 with isolated chondrocytes and measurement of gene expression in the presence or absence of PG synthase inactivation by siRNA may be the best way to differentiate between PGE_2_-dependent and PGE_2_-independent signaling in cartilage protection. In preliminary studies (E. Tchetina, J. DiBattista, A.R. Poole, unpublished data) we have observed that the addition of Naproxen, a PG synthase inhibitor, can abrogate the inhibitory effect of TGF-β2 and enhance collagen cleavage in the absence of this growth factor. This also suggests that PGE_2 _may have a role in mediating the action of TGF-β2 and in protecting articular cartilage from degeneration.

Some variability was observed in the effect of PGE_2 _in individual patients. This may have been due to differences in the phenotypic expression of chondrocytes associated with the degree of cartilage degeneration, which varies between and within patients. This is an issue that we were unable to address in the present study because a lack of tissue at arthroplasty forced us to pool all the cartilage from each patient. Differences between patients in response to agents that can regulate matrix resorption have been noted previously in culture studies of this kind [3,18]. Further investigations are clearly required to help explain these differences. However, the present results do support our previous suggestion that increased generation of PGE_2 _is associated with the predominantly inhibitory effect of TGF-β_2 _on the collagen degeneration in OA articular cartilage [3] and that PGE_2 _can suppress collagen cleavage.

PGE_2 _was previously found to suppress chondrocyte differentiation/hypertrophy in development [24,25]. Here we show that this inhibitory effect on hypertrophy can be extended to the pathology of OA cartilage. Chondrocyte hypertrophy, defined by the presence of COL10A1 expression, is a recognized component of the pathology involving excessive collagen cleavage. Both are frequently suppressed in our studies by very low concentrations of added PGE_2_. The fact that added PGE_2 _is capable of suppressing the chondrocyte differentiation/hypertrophy phenotype in development [24,25], and that in our explants 10 pg/ml PGE_2 _also inhibits chondrocyte terminal differentiation (registered by COL10A1 downregulation), suggests that PGE_2 _controls collagen cleavage by suppressing the increase in collagenase activity associated with chondrocyte hypertrophy [1-3]. The simultaneous suppression of proinflammatory cytokines that we observed also suggests that the upregulation of IL-1 and TNF-α is associated with hypertrophy, at least in OA. In this respect PGE_2 _is again very similar to TGF-β2 in downregulating chondrocyte hypertrophy and restoring the healthy early proliferative chondrocyte phenotype [3].

That chondrocyte hypertrophy is associated with cartilage degeneration in OA is also supported by the fact that partial deletion of *RUNX-2 *gene expression, which is believed to account for chondrocyte hypertrophy and MMP-13 upregulation (the two events being closely linked), retards cartilage degeneration in a mouse surgical model [26]. Downregulation of excessive collagen cleavage in OA by suppression of expression of a hypertrophic phenotype could therefore be a promising approach in controlling the disease severity.

These suppressive effects of PGE_2 _were seen only at much lower concentrations than those observed in inflammation [7]. PGE_2 _has been reported to be responsible for both catabolic and anabolic changes in articular cartilage [6,7]. It can inhibit collagen synthesis in growth plate chondrocytes [27], mediate MMP production in articular chondrocytes and cartilage [6,28], inhibit chondrocyte proliferation [29], promote IL-1β expression [30] and induce chondrocyte apoptosis [31]. In contrast, it has also been shown to inhibit collagenase and stromelysin expression [9,10,32,33] and IL-1β and TNF-α generation [33], to stimulate collagen and proteoglycan synthesis [11-13] and chondrocyte proliferation [11], and to inhibit chondrocyte terminal differentiation/hypertrophy [24,25] and apoptosis in endothelial cells [34].

It is important to recognize that the opposing effects of PGE_2 _on chondrocytes can be dependent on the dose of the agent [9,35]. Our experimental system containing added 10 pg/ml PGE_2 _produced chondroprotection on OA articular cartilage in that it downregulated collagen cleavage and the expression of genes associated with collagen cleavage, proinflammatory cytokines and hypertrophy. It did not upregulate chondrocyte proliferation and type II collagen synthesis as evidenced by the lack of significant changes in the expression of cyclin B2 and COL2A1, respectively. Generation of prostaglandins was also not altered as COX-2 and PGES-1 expression remained essentially the same. The reasons why only such low doses of PGE_2 _are chondroprotective remain to be established but may be related to receptor usage.

It is well established that EP_1 _and EP_2 _receptors for PGE_2 _are low-affinity receptors, whereas EP_3 _and EP_4 _are high-affinity receptors. Activation of the low-affinity PGE_2 _receptors is likely to be important in mediating the actions of the much higher concentrations of PGE_2 _found in various pathologic inflammatory processes [36]. Simultaneous stimulation of EP_2 _and EP_4 _is necessary and sufficient to elicit the effect of PGE_2 _on the differentiation of rat primary chondrocytes [37]. Receptor deletion studies have shown that homozygous deletion of the EP_1_, EP_2 _or EP_3 _receptors does not affect the development of arthritis, whereas EP_4 _receptor-deficient mice showed decreased incidence and severity of disease [38]. In contrast, others have found that suppression of EP_2 _expression enhances MMP-13 collagenase induction in human OA chondrocytes [39].

Our own preliminary data on receptor involvement suggest a role of EP_3 _receptor, which expression is significantly upregulated on the addition of 10 pg/ml PGE_2 _to OA articular cartilage explants (E.V. Tchetina, J. DiBattista, A.R. Poole, unpublished data). Further extensive work to identify the receptors involved is therefore required to help explain our observations.

Finally, it is important to recognize the fact that in the presence of additional physiological amounts of PGE_2_, not only are OA chondrocytes often capable of reverting to a more normal phenotype, as reflected by studies of gene expression and matrix collagen degradation, but osteoblastic differentiation, detected by alkaline phosphatase activity, is also enhanced [35,40]. The present results, although preliminary, suggest the importance of very low concentrations of PGE_2 _in maintaining a healthier skeleton and specifically a more normal chondrocyte phenotype in ageing articular cartilage.

## Conclusion

We have shown that PGE_2 _at low concentrations in OA cartilage can downregulate collagenase-dependent COL2A1 cleavage and the associated hypertrophy. This was associated with a downregulation of collagenases MMP-13 and MMP-1, proinflammatory cytokines IL-1β and TNF-α, and COL10A1 expression. Proteoglycan degradation was not affected. Therefore PGE_2 _at concentrations much lower than those generated in inflammation are chondroprotective and not destructive as is commonly believed.

## Abbreviations

COL10A1 = type X collagen; COL2A1 = type II collagen; COX = cyclooxygenase; DMEM = Dulbecco's modified Eagle's medium; ELISA = enzyme-linked immunosorbent assay; GAG = glycosaminoglycan; GAPDH = glyceraldehyde-3-phosphate dehydrogenase; IL = interleukin; MMP = matrix metalloproteinase; OA = osteoarthritis; PCR = polymerase chain reaction; PG = prostaglandin; PGE_2 _= prostaglandin E_2_; PGES-1 = prostaglandin E synthase-1; TGF-β2 = transforming growth factor-β2; TNF-α = tumor necrosis factor-α.

## Competing interests

The authors declare that they have no competing interests.

## Authors' contributions

EVT planned the work, performed all the laboratory experiments and performed the statistical analysis as well as drafting the manuscript. JADB assisted in the experimental design and interpretation of data and helped to draft the manuscript. DJZ and JA assisted in the experimental studies. ARP participated in the design of the study and helped to draft the manuscript. All authors read and approved the final manuscript.
